# Influence of Different Vinification Techniques on Volatile Compounds and the Aromatic Profile of Palomino Fino Wines

**DOI:** 10.3390/foods10020453

**Published:** 2021-02-19

**Authors:** Ana M. Roldán, Fini Sánchez-García, Luis Pérez-Rodríguez, Víctor M. Palacios

**Affiliations:** Department of Chemical Engineering and Food Technology, Faculty of Sciences, University of Cadiz, Box 40, 11510 Puerto Real, Cadiz, Spain; fini.sanchez@uca.es (F.S.-G.); luis.perez@uca.es (L.P.-R.); victor.palacios@uca.es (V.M.P.)

**Keywords:** pellicular maceration, supra-extraction, β-glycosidase, enzymes, yeasts, volatile compounds, sensory analysis

## Abstract

The aim of this study was to evaluate the influence of vinification techniques on volatile compounds and sensory profiles in young Palomino fino white wines. Four winemaking techniques (pellicular maceration, supra-extraction and use of commercial yeast strains and of β-glycosidase enzymes) were implemented to enhance the aromatic quality of wines elaborated from this neutral variety of grape. Volatile compound content, aromatic profile (OAVs) and sensorial analysis were determined. The results showed that all the vinification techniques studied led to an increase in volatile compounds compared to the control wine. Likewise, an influence of the vineyard and must extraction method on these compounds was observed. However, the greatest changes in aroma activity and sensory profile were a result of the pellicular maceration and supra-extraction techniques. The latter was differentiated by the highest content of terpenes and, consequently, the highest odour activity values of floral series. In addition, the supra-extraction was a very selective technique since it extracted terpenes and aromatic precursors, but not the acids responsible for the fatty characteristic, such as octanoic acid. In terms of sensory profile, the supra-extraction technique improved the intensity of the Palomino fino white wine and its aromatic quality with a previously not-determined floral character.

## 1. Introduction

Palomino fino is the undisputed leading grape variety in the Jerez-Xeres-Sherry D.O. wine production area (Andalusia, Spain) and is considered a key element in the production of dry and sweet sherry wines by biological and oxidative aging [1]. This grape variety is adapted to the warm conditions of this south-western Spanish region [2], and although it is characterized by its high yield, no remarkable aromatic attributes have been found [3]. In fact, the Palomino fino grape variety is considered a “neutral” grape with low content of aroma precursors [2]. This “neutral” characteristic makes this grape variety ideal for sherry wine production. This is because its chemical composition, aroma and sensory characteristics are determined by biological and chemical processes that take place during aging [4] (during the dynamic phase in the “solera” system), but not for fresh and fruity white wines. In addition, the low total acidity of these wines produced in a warm climate contributes to the minimal aromatic intensity and also to the lack of freshness [5]. 

In order to diversify traditional production by adapting it to current needs or demands, in recent years, the use of the Palomino fino variety has been promoted for the production of young white wines. Therefore, it is necessary to implement alternative cultivation and technological practices in order to intensify the aromatic potential of these wines. It is remarkable that the aroma of wine constitutes an important factor in the wine quality and price as well as a preference attribute for consumers [6,7]. The variety of grape employed in making a particular wine, in many cases, completely determines the aroma of that wine [8,9,10]. However, it is well known that other factors such as climate, region, viticultural practices, degree of grape maturation, yeast and winemaking techniques, including aging, influence the wine aroma [11,12,13,14,15,16,17,18]. Among them, pre-fermentative cold maceration (cold soaking or cryomaceration) and the application of selected yeasts and pectinases are examples of techniques used by oenologists to improve the aromatic quality and sensory characteristics of white wines. The first technique leads to the greater extraction of aromatic compounds and precursors from the skins by means of maceration in the must before the fermentation [19]. The use of yeasts and enzymes with glycosidase activity favour the release of the aromatic compounds that are found in the grape combined with sugars in non-aromatic form [20,21]. 

Some studies have been published on the influence of certain winemaking techniques on the volatile compounds of Palomino fino wines and other warm-climate grape varieties (Jerez region, Andalusia, Spain) and comparisons between them. These winemaking techniques include cold soaking [8], addition of glycosidase enzymes [5,8], co-inoculation of non-*Saccharomyces* and *Saccharomyces cerevisiae* yeast [2] and the use of bee pollen as a fermentative activator [22]. However, although some authors considered this variety to have good potential for producing new styles of wines [2], it is not clear if the aroma and quality of the Palomino fino wine is improved or which of the winemaking techniques used offer the best results. Therefore, the aim of this study was to assess the effect of various vinification techniques (pellicular maceration, supra-extraction (freezing and thawing of grapes before pressing) and the use of different types of yeast strains and β-glycosidase enzymes on volatile compounds and sensory profiles in white wines elaborated from an autochthonous grape variety: Palomino fino.

## 2. Materials and Methods 

### 2.1. Winemaking Techniques 

Palomino fino grapes and must from vineyards and wineries, respectively, belonging to the Jerez area (Cádiz, Spain, coordinates: 36.6866, −6.13717) and from two vintages (2006 and 2007) were used to perform the trials. 

#### 2.1.1. Pellicular Maceration and Supra-Extraction Trials

Palomino fino grapes for pellicular maceration (PM) and supra-extraction (SUPRA) were from the “CIFA Rancho La Merced” in Jerez de la Frontera (Cádiz, Spain). Grapes were transported in 25 kg food-material boxes to the laboratory, where they were separated by weight into 5 equal fractions of 10 kg each. One fraction of whole grapes was frozen at −18 °C until its use in SUPRA trials, and the rest was destemmed and ground and must and skins were separated. Subsequently, 4 mixtures of 1:3 must and skins were prepared. Each mixture was kept in a refrigerated tank of 5 L capacity where macerations were performed at different maceration times: 0 h (control), 4 h (PM4), 8 h (PM8) and 12 h (PM12) and at a controlled temperature (15 °C). A total of 8 tanks were used to carry out the trials in duplicate. After maceration, pressing of each tank was performed and potassium metabisulfite (50 mg/L) and tartaric acid were added to the musts to correct their pH to a value of 3.5–3.6 (both Agrovin, Ciudad Real, Spain). The sulphited grape musts were then subjected to static settling for 24 h at a low temperature (10 °C). The clarified grape musts were then racked to glass tanks with cooling jackets (V = 5 L) for directed alcoholic fermentation at a controlled temperature of 18 °C using a commercial active dry wine yeast (ADWY) strain of *Saccharomyces cerevisiae* Fermivin 7013 (DSM Food Specialties Spain, S.L., Barcelona, Spain), at a dose of 20 g/hL. This ADWY is recommended for white and red wines and is characterized by neutrality and fast fermentation. Once the alcoholic fermentation was complete (stable density measurement and the residual reducing sugars below 2 g/L), the wines were chilled (6 °C) for 7 days and subsequently treated with gelatin (4 g/hL) and bentonite (40 g/hL), filtered (sterilizing filter plates SA-990) (Papeleras del Besós Placas filtrantes, S.L., Barcelona, Spain) and bottled using nitrogen pressure. 

For the SUPRA trials, once the grapes were thawed and pressed, the same winemaking protocol was followed as for the PM.

#### 2.1.2. Yeast Strains and Enzyme Trials

The Palomino fino grape must, without the addition of sulphurous anhydride and pH correction, was obtained from the Grupo Osborne winery in El Puerto de Santa María (Cádiz, Spain). Sulphurous addition, pH correction and settling of the musts were carried out in the same way as in the PM and SUPRA trials. Once the grape must was clarified, it was separated into 4 fractions and racked to the fermentation tanks. A total of 8 tanks were used to carry out the trials in duplicate. Four yeast strains (3 commercial and 1 selected native) were used to carry out the alcoholic fermentation by inoculating each tank with each yeast strain. A total of 8 tanks were used to carry out the trials in duplicate. In addition, Fermivin (DSM Food Specialties Spain, S.L., Barcelona) (F) at a dose of 20 g/hL (used as reference), ENSIS-L5 (Ensis Sciences, Barcelona, Spain) at a dose of 10 g/hL (ENSIS) and CK S-102 (Enolviz, S.L., Bilbao, Spain) (CK) at a dose of 15 g/hL were used. The fourth strain was an autochthonous strain of *S. cerevisiae* isolated by the winery Domecq S.L. in Jerez de la Frontera (Cádiz, Spain) and commonly used as “pie de cuba” (PC) in the elaboration of fino-type wines. 

After complete fermentation, each type of wine (elaborated by each yeast strain) was separated into 3 new fractions. One fraction was clarified, filtered and bottled following the same process as the PM and SUPRA wines obtaining the PC, F, ENSIS and CK wines. The second and third fractions of each wine were kept in clarification tanks for 2 weeks at 20 °C after the addition of a commercial enzyme extract with high β-glucosidase activity to each fraction. The enzyme extracts were Novoferm 12G (Novo Nordisk Pharma S.A., Madrid, Spain) (12G) and Rapidase AR-2000 (DSM Food Specialties Spain, S.L., Barcelona, Spain) (AR) at a dose of 4 and 2.5 g/hL, respectively. The β-glucosidase activity under the assay conditions was determined, resulting in 33.7 and 38.6 units/g of extract for 12G and AR, respectively. After enzymatic treatment, the wines were clarified, filtered and bottled, obtaining 8 types of wine (PC-12G, PC-AR, F-12G, F-AR, ENSIS-12G, ENSIS-AR, CK-12G and CK-AR).

### 2.2. Volatile Compounds 

Higher alcohols, acetaldehyde, ethyl acetate and methanol were analysed using a GC-FID HP 5890 Series II system (Agilent Technologies, California, USA) equipped with a Carbowax 20 M column (50 m, 0.25 mm ID, 0.25 μm). The operation conditions of the GC were: injector and detector temperatures, 250 °C; oven temperature, 35 °C for 10 min (followed by a ramp of 4 °C/min until 200 °C); sample volume, 20 µL (distilled sample for alcoholic strength determination) in split mode (split ratio 1/20); and carrier gas, H (1 mL/min). The identification and quantification of major volatile compounds was carried out using pure standard compounds (Sigma–Aldrich Química, S.A., Madrid, Spain) and 4-methyl-2-pentanol (783 mg/L) as the internal standard. 

Semiquantitative GC–MS analyses, after solid-phase extraction (SPE), were used to determine minor volatile aroma compounds. The method described by Di Stefano [23] was followed for the extraction using DSC-18 of 1 g (6 mL) cartridges (SUPELCO, Bellefonte, PA, USA). GC–MS analysis was performed on a Voyager^®^ system (Termoquest, Milan, Italy), equipped with a Supelcowax-10 column (60 m, 0.32 mm ID, 0.5 μm). The injector and detector temperature was 200 °C, using He (1 mL/min) as the carrier gas. The GC oven program was as follows: held at 40 °C for 5 min, then ramped at 2 °C/min to 200 °C and held for 5 min. A direct injection of 2 μL in splitless mode (40 s) of sample was carried out. The electronic impact mode (EI+) with an electron energy value of 70 eV was applied. The initial and interface temperatures were 220 °C and 320 °C, respectively. The MS collected data at a scan index of 1 scan/s and mass acquisition range of 45–400 *m*/*z*. The procedure was also described in [22]. Peak identification was carried out using the Xcalibur v.1.1. Library Browser (Thermo Fisher Scientific, Waltham, MA, USA) by analogy of mass spectra (90% minimum matching level), some of them confirmed by retention times of standards from Sigma Aldrich (St. Louis, MO, USA). Semiquantitative analyses were carried out, assuming a response factor equal to one. All determinations were carried out in duplicate. Total volatile compounds were determined by the sum of the compounds identified and quantified.

### 2.3. Odour Activity Values 

According to Francis et al. [24], odour activity values (OAV) were calculated as the ratio between the mean concentration of each volatile aroma compound and its odour threshold value (OTV), as reported by other authors [25,26,27,28,29,30]. To estimate the overall wine aroma, the odour descriptors were grouped into different aromatic series and every compound was assigned to one aromatic series based on the main odour descriptors. The total intensities for each aromatic series were calculated as the ΣOAV of each of the compounds assigned to this series. The odorant series used in this study (fruity, sweet, fatty, floral, grassy, spicy, earthy and mushroom, chemical and dried fruit) represented the main constituents of the aromatic profile used by Amores-Arrocha et al. [22] for the wines made using the same grape variety. An organoleptic profile of the wines was obtained through the relationship between quantitative results derived from chemical and sensory analyses [31].

### 2.4. Sensory Analysis 

The differences between the sensorial profiles of wines made using different winemaking techniques and the control wine were evaluated by a panel of 10 trained tasters, both men and women between 30 and 55 years of age. All wines were evaluated between three and five days after bottling. The tasting panel used individual booths with controlled illumination, located in the tasting room of the Institute of Viticulture and Agri-Food Research (IVAGRO, Puerto Real, Cádiz); wines were presented in standard tasting glasses [32] and covered with watch-glasses to minimize the evaporation of volatile compounds. The wines were served to each taster (50 mL) at room temperature (20 ± 2 °C). A 5-point scale (from 0 to 5 according to increasing intensity) was used to evaluate the general acceptability of wines and their visual (intensity and quality), olfactory (quality and intensity) and gustatory (quality and intensity) characteristics. Some aspects of interest such as characteristic odour (floral, fruity, vegetable, spice, balsamic and dried fruit), flavours (acidity, salinity, sweetness, bitterness and warmness) and mouthfeel (smoothness, persistence and aftertaste) were also considered following the procedure by Amores-Arrocha [22]. The tasting descriptors used were selected based on those for white wines defined by Jackson [33]. 

### 2.5. Statistical Analysis 

A two-way analysis of variance (ANOVA) was performed to identify statistically significant differences between samples using the statistical package GraphPad Prism version 6.01 for Windows (GraphPad Software, San Diego, CA, USA). Statistically significant differences between samples according to Bonferroni’s multiple range (BSD) test was defined as *p* < 0.05. A principal component analysis (PCA) was performed to determine the influence of the vinification techniques on the aromatic profile of wines. The statistical computer package SPSS 23.0 (SPSS Inc., Chicago, IL, USA) and the factor extraction method of rotated component matrix loadings were used, with “quartimax” Kaiser normalization. 

## 3. Results and Discussion

### 3.1. Influence of Pre-Fermentative and Aroma-Release Treatments on Volatile Compounds of Wines

**Figure 1:**
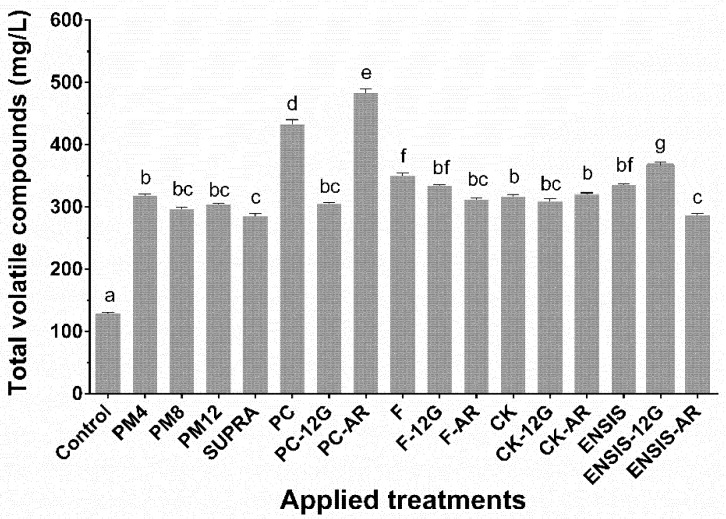
Total volatile compounds in Palomino fino wine samples with application of extractive and aroma-release techniques. Lowercase superscript letters show statistical significance (*p* < 0.05). PM4, PM8 and PM12: pellicular maceration for 4, 8 and 12 h, respectively; SUPRA: supra-extraction wine; PC, F, CK and ENSIS: wine elaborated by different yeast strains; 12G and AR: correspond to enzymatic extracts used. PC: “pie de cuba”; F: Fermivin; CK: CK S-102; ENSIS: ENSIS-L5; 12G: Novoferm 12G; AR: Rapidase AR-2000.

Total volatile compounds of the Palomino fino control wine and those obtained by the different techniques are shown in Figure 1. As can be observed, the PM and SUPRA techniques led to an increase of the total volatile compounds similar to those produced by most yeast strains or glycosidase enzymes. 

It is necessary to highlight that the control and F wines were made with the same yeast strain and under the same conditions, but the grapes and must came from different vineyards (Jerez and El Puerto de Santa María, respectively) and were obtained with different pressing systems (horizontal membrane presses and horizontal plate presses, respectively). The differences observed in the total volatile content between both were conditioned by both factors. Several authors [10,34] have shown the influence of terroir and grape ripening [10,12] on volatile compounds, as well as influence of technological practices [8,13]. Also, it has been reported that the greatest extraction of grape solids is obtained by application of a high degree of pressure, which modifies the physicochemical and sensory composition of the wines [35,36]. However, to date, no studies have been carried out about the influence of press type on volatile compounds. According to these results, horizontal membrane presses lead to lower total volatile compounds than horizontal plate presses, most likely due to less extraction from the solid parts, particularly aromatic compounds present in the grapes. On the one hand, the PM and SUPRA favoured this extraction, increasing total concentrations of most aromatic components in the final wine [37]. According to Ribereau-Gayón et al. [38], supra-extraction causes changes in the ultrastructure of the grape tissues, producing an effect comparable to that of skin maceration and releasing aromas and aromatic precursors more easily from the grape. Therefore, winemaking practices that favour grape crushing and extraction of components from the skins (including pressing) lead to wines with a higher content of volatile compounds.

On the other hand, the total content of the volatile compounds found in Palomino fino wine was also dependent on the yeast strain used. The maximum proportion of volatile compounds was yielded by the PC strain, followed by the F strain, whereas similar levels of volatiles were found in the CK and ENSIS wines. The PC strain is an autochthonous yeast strain used in sherry wine elaboration characterized by higher alcohol and acetaldehyde production, hence the higher production of volatiles. 

Regarding β-glycosidase enzymes, an increase of total volatile compounds was observed with respect to the control. However, their effects were reflected less clearly, being dependent on the characteristics of the wine after fermentation with a specific yeast strain. 

The 46 volatile compounds positively identified and quantified in Palomino fino white wines obtained with pre-fermentative and aroma-release treatments are shown in Table 1 and Table 2, respectively. The compounds are grouped according to their chemical structure, including higher alcohols, methanol, acids, C6 alcohols, alcohols, terpenes, esters, aldehydes, thiols and phenols. As can be seen, the higher alcohols, methanol, esters and acetaldehyde produced an enrichment in the volatile compounds of wines made with different vinification techniques.

#### 3.1.1. Higher Alcohols and Methanol

As can be seen from the results in Table 1 and Table 2, higher alcohols were the largest group of volatile compounds in the evaluated Palomino fino wines, in agreement with results reported by other authors for this and other white wine varieties [6,22,39]. Among them, the isoamyl alcohol was quantitatively the main compound, reaching levels above 200 mg/L in yeast strain- and enzyme-treated wines regardless of the yeast strain and β-glycosidase enzyme used. It was reported that the greatest amounts of higher alcohols are obtained from wines fermented with the highest level of solids [40], which could explain these results. 

The pellicular maceration and supra-extraction favoured the formation of higher alcohols, with a higher influence of the processes themselves (Table 1) on this increase than the time of maceration, which could be related to the amino acid content on which the pellicular maceration exerts the same effect [41]. 

As can be seen in Table 2, the relative concentrations of total alcohols in yeast strain- and enzymatically treated wines were significantly higher than those of the control and similar between them. They are the main yeast-synthesised aroma substances of the fermentation bouquet by sugar catabolism or decarboxylation and deamination of amino acids in yeast [42], which would explain the differences between the yeast strains used.

Similarly, the methanol content of all wines also increased, generally reaching levels twice that of the control. This increase could be due to the presence of the high pectin content in must. Pectic residues, mainly cellulose and pectins, are released from the cellular tissue due to the damages, breaks and maceration. The yeast could solubilize and metabolize pectin to produce methanol during alcoholic fermentation by enzymatic hydrolysis [43]. In terms of alcohol production and food safety, pellicular maceration may not be recommended. However, the levels produced by this technique are well below the limits established by the International Organisation of Vine and Wine (OIV) for white wines (250 mg/L) in its resolution No. OENO 19/2004 [44].

#### 3.1.2. Acids 

A total of six acids were identified in the volatile fraction of Palomino fino wines. However, in yeast strain- and enzyme-treated wines, only hexanoic, octanoic and *n*-decanoic acids were detected and quantified. The average total concentration of these acids ranged between 0.4 and 1.9 mg/L, reaching the highest values in the control and PM wines. Therefore, they could contribute to the quality of the wine by increasing aroma complexity [6].

On the one hand, the octanoic acid, followed by hexanoic acid, showed the highest contribution to the total acid content (Table 1 and Table 2). The *n*-decanoic, 9-decenoic and benzoic acids were present in the control, PM and SUPRA wines. The first two generally increased with maceration process and time, while benzoic acid decreased until 50 µg/L and was not detected in SUPRA wines (Table 1). 

On the other hand, it has been reported that the production of fatty acids is dependent on the yeast strains and their abilities to synthesize/convert precursors or break carbon chains [45]. These varying abilities could explain the differences in acids observed when different yeast strains were used (Table 2). The CK and F wines showed the highest content in fatty acids, highlighting octanoic acid (>500 µg/L), while PC wines showed the lowest content. Likewise, although no references have been found, treatment with glycosidase enzymes also affected the acid content, but the behaviour of each enzyme differed according to the strain. So, the F and PC wines treated with 12G showed higher concentrations of fatty acids than the same untreated wines, while the opposite effect was observed in CK and ENSIS wines. However, the AR enzyme treatment led to lower content of fatty acids than their respective untreated wines. 

#### 3.1.3. C6 Alcohols

C6 alcohols are known to contribute to the aroma of many fruits and vegetables [46] and have also been proposed as potential markers of varietal authenticity [47]. Some authors [48,49] have even described them as major contributors to the varietal aroma of neutral grape varieties. Besides, according to Denis et al. [50], hexan-1-ol, hexenal, (E)-2-hexen-1-ol and (E)-2-hexenal are all precursors to hexyl acetate, although their metabolization by yeast depends on the concentration and type of C6 compounds. On the one hand, the C6 alcohol levels of Palomino fino wines varied according to the treatment used, with 1-hexanol as a highlight (Table 1 and Table 2). The SUPRA wines (Table 1) showed the lowest C6 compounds’ content because of the low levels of 1-hexanol and also (E)-3-hexen-1-ol (27.83 and 0.25 µg/L, respectively). On the other hand, the wines elaborated by the PC strain (Table 2) showed lower contents of these compounds, principally (E)-3-hexen-1-ol, while no significant effect of enzyme treatment was observed. Bakker et al. [51] pointed out that C6 compounds are originally present in crushed grape must, resulting from the enzymatic oxidation of grape polyunsaturated fatty acids through the lipoxygenase pathway. In this sense, extractive treatments did not favour the yield of C6 alcohols in our study, contrary to what was observed in other grape varieties [14,37,41]. 

#### 3.1.4. Alcohols

As shown in Table 1 and Table 2, the type of technological treatment applied also had an impact on the alcohol content of wines, ranging between 0.5 and 2 mg/L. Phenylethyl alcohol was the principal contributor to this content, and the SUPRA wines showed the lowest level. 

Only 1-octanol was detected in the control, PM and SUPRA wines, while 3-ethoxy-1-propanol, 3-ethyl-2-pentanol and benzyl alcohol were present in the other wines. Likewise, each alcohol’s formation and its content in wine depended on the yeast strain and enzyme used. The highest levels were obtained with the CK strain and the lowest with the PC. Glycosidase enzymes showed different behaviour depending on the wine characteristics. The AR enzyme increased the alcohol content in the F, PC and ENSIS wines, and 12G had a stronger effect on the F and PC wines. Therefore, alcohol contents were dependent on the raw material and the biochemical and technological changes during winemaking, as indicated by some authors [52].

#### 3.1.5. Terpenes

Terpenes are considered as the main part of the varietal compounds derived from grapes, where they are often found to be glycosidically bound [42], depending on the variety and the relative proportions of free and bound forms [52]. The presence of terpenes in wine is typically due to the direct extraction of these compounds and their skin glycoconjugates [14,42,53]. However, during fermentation, terpene glycosides are hydrolysed to free volatile terpenes by yeast glycosidase and by the acidic fermentation conditions.

According to Genovés et al. [54], 2,6-dimethyl-3,7-octadiene-2,6-diol is, together with geraniol, one of the major terpene compounds in Palomino musts. Other terpene compounds, such as linalool, nerol and a-terpineol, have been found for the same variety by some authors [5,8]. However, as can be observed in Table 1 and Table 2, the content of terpenes in Palomino fino wines is represented by 2,6-dimethyl-3,7-octadien-2,6-diol and a small fraction of β-citronellol, except in the SUPRA wines (Table 1). In fact, the latter showed a terpene content higher than 200 µg/L, where 2,6-dimethyl-3,7-octadiene-2,6-diol was the majority terpene compound. On the one hand, this technique favoured the extraction of other terpenic compounds, such as linalool, linalool oxide, α-terpineol, β-citronellol and nerol, or their precursors. On the other hand, no increase in terpenes due to pellicular maceration and the use of yeast strains or glycosidase enzymes was observed, contrary to other studies [5,8,55]. In general, the terpene content was similar, except when compared to the PC wines (12–15 µg/L), which showed the lowest contents. Therefore, this autochthonous yeast strain generally used for sherry wine elaboration seems to have a lower capacity to release glycosidic compounds. The content of aromatic precursors extracted from the grape, their presence in the must and the glycosidase activity of the yeasts during fermentation conditioned the release of glycosylated terpenes [56]. 

The disruption of membranes by freezing [57] produced during the supra-extraction favours the selective extraction of precursors and aromatic compounds and their subsequent diffusion into the must, even in varieties such as Palomino fino, which is considered neutral by some authors [54]. 

#### 3.1.6. Esters

Esters were found in a range between 8.0 and 63.0 mg/L (Table 1 and Table 2), showing the higher contents (>24.0 mg/L) in wines made with different yeast strains and glycosidase enzymes, most likely due to the must characteristics used in winemaking. The biosynthesis of these compounds is directly dependent on the availability of fatty acids, and both depend on fermentation conditions such as yeast strain, nutrient status of the must (e.g., sugar, assimilable nitrogen) and temperature [58]. On the one hand, the SUPRA and PM techniques increased the total esters, but no influence of maceration time was observed. Regarding the yeast used, a greater influence of the must composition was observed compared to the yeast strain. On the other hand, the β-glycosidase enzymes used led to a lower content of esters, contrary to what has been shown in other studies for the same variety [2,5]. The observed decrease was greater with the use of 12G, except for the ENSIS wines. This fact shows that both preparations possess some type of secondary hydrolytic activity that mainly affects these esters [59]. 

The compound responsible for the total ester content and the differences found between Palomino fino wines was ethyl acetate, with the highest concentration. The wines elaborated using the PC strain stood out due to their levels of ethyl acetate, but the use of enzymes caused a decrease of this compound. Significantly high levels (>50.0 µg/L) of ethyl pentanoate, ethyl octanoate, ethyl decanoate, diethyl succinate, phenethyl acetate and ethyl 9-decenoate were found in these wines, although this last did not reach higher values than 16.0 and 10 µg/L in the F and PC wines, respectively. The behaviour of these esters depended on both the technique used and the compound itself. The pellicular maceration favoured the formation of ethyl octanoate, whose content increased with time. The SUPRA wines were characterized by their isoamyl acetate, ethyl pentanoate and ethyl octanoate content. The differences observed in the content of these esters in wines made with different yeast strains were due to the formation and concentration of the yeast used in the present study, but similar levels were reached with all of them except the PC yeast strain. Similar findings have been reported by several other authors [60] about the influence of *S. cerevisiae* on ester formation. 

According to Amores-Arrocha et al. [22], the ester levels observed could be explained by the presence of fatty acids in wines, because their synthesis is conditioned by the greater or lesser content of fatty acids and alcohols, both substrates of the esterification reactions. 

#### 3.1.7. Acetalehydes

Acetaldehyde is formed mainly by the metabolism of yeasts [6], but also by acetic acid bacteria, andcoupled auto-oxidation of ethanol and phenolic compounds [61]. This aroma compound is one of the most important sensory carbonyl compounds formed during vinification [61] and is associated with fruity aromas and notes of nuts or dried fruits [6]. As can be observed from the results in Table 1 and Table 2, significant differences were observed between the studied wines. Acetaldehyde production differed significantly between the two types of must, ranging from 11 to 18 mg/L and 30 to 150 mg/L in wines obtained by extractive and aroma-release techniques, respectively. The control and the PM4 and SUPRA wines showed similar levels of acetaldehyde, but longer maceration times led to a decrease. Various factors influence the formation of acetaldehyde, including the medium composition [62]. These results show that the ability to produce acetaldehyde is a property of yeasts and that the *S. cerevisiae* strains used produce relatively higher levels of acetaldehyde than those described by other authors [62,63]. Therefore, the yeast strain represents a prominent factor in determining the content of acetaldehyde in wine distinguishing between two different phenotypes: high and low acetaldehyde producers [63]. In this sense, the PC strain’s ability to produce acetaldehyde is worth highlighting, since it could be considered a high acetaldehyde producer; hence the interest in its use for sherry winemaking, where acetaldehyde is a well-known and desirable constituent [64].

The glycosidase enzymes had a different effect on acetaldehyde content. The 12G enzyme gave rise to lower acetaldehyde content, except in the ENSIS wines, while AR increased this content. No studies were found on the effect of the use of enzymes on the acetaldehyde content in wines. However, the accumulation of acetaldehyde during fermentation has been reported to be dependent on the equilibrium between the alcohol, dehydrogenase and aldehyde dehydrogenase enzymes [65]. The residual activity of these enzymes still present in the wine or of the enzyme extracts used could be responsible for the results obtained. 

#### 3.1.8. Other Compounds

Other compounds, such as thiols and phenols, were found in exceptionally low concentrations in some of the Palomino fino wines. These compounds were 3-methylthio-1-propanol, 2-methoxy-4-vinilphenol and 2,6-di-*tert*-butyl-4-ethylphenol, representing under 0.1% of the total amount of volatile compounds. The first was found in all the elaborated wines, ranging from 2 to 9 µg/L, while 2-methoxy-4-vinilphenol was found in the control and PM wines and its content increased with maceration time. Only the control, PM and SUPRA wines showed 2,6-di-tert-butyl-4-ethylphenol, with remarkably similar concentrations, except in the latter with the lowest content (5.8 µg/L). 

### 3.2. Odour Activity Values (OAVs) and Aromatic Series

Odour activity values (OAVs) were used to estimate the sensory contribution of the aromatic compounds to the overall flavour of the wines. The contribution of one specific volatile compound to the perception of the aroma depends not only on the concentration of the volatile compound itself, but also on its odour threshold value. In this regard, the odour perception threshold (OPT) value, odour descriptors and odorant series (by the main odour descriptor) of the volatile compounds found in Palomino fino wines were revised and listed in Table 3. According to Sánchez-Palomo et al. [6], compounds present in both higher and lower concentrations compared to their odour threshold were included; the latter due to synergistic effects with other odorant compounds. 

**Figure 2:**
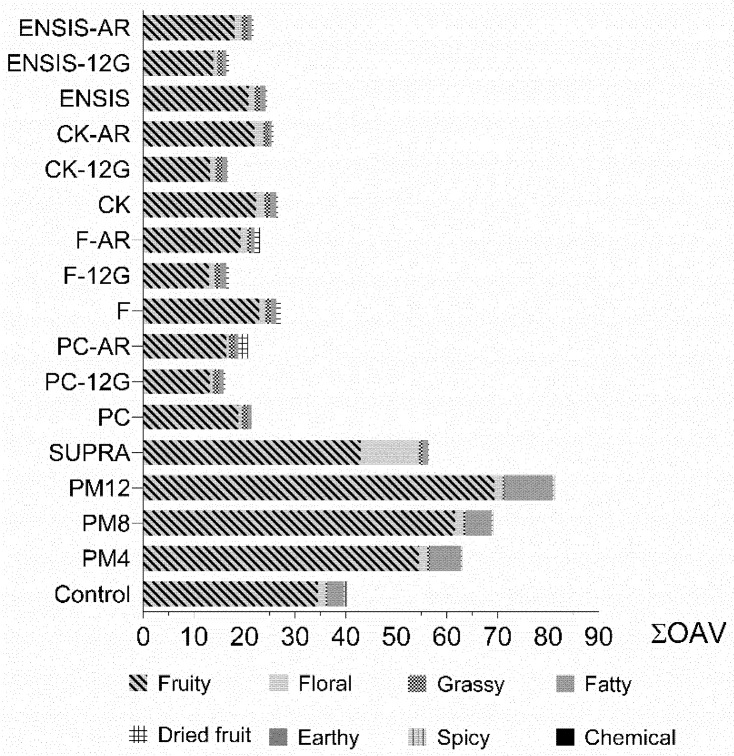
Summary of odour activity values (ΣOAV) by odorant series. PM4, PM8 and PM12: pellicular maceration for 4, 8 and 12 h, respectively; SUPRA: supra-extraction wine; PC, F, CK and ENSIS: wine elaborated by different yeast strains; 12G and AR: correspond to enzymatic extracts used.

Considering the ΣOAV by odorant series (Figure 2) and the total OAV, two clearly differentiated groupings in the wines are observed: on the one hand, the control and wines treated with extractive techniques whose musts were obtained by horizontal membrane pressing; and on the other hand, the wines elaborated using different yeast strains and glycosidase enzymes to release aroma, whose musts were obtained by horizontal plate pressing. Considering the control and F wines, both fermented by the same yeast strain, although the total volatile content was higher in the F wines, their ΣOAV was lower and the opposite occurred in the control wines. Therefore, the total volatile content was not directly related to the aromatic profile given by the compounds contributing to aroma. 

Fruity was the predominant odorant series contributed by esters, principally isoamyl alcohol and ethyl octanoate. These compounds have a low perception threshold, so contributed more significantly to the OAV. Their values increased with the pellicular maceration time, reaching maximum levels at 12 h. The time of contact with the skins favoured the grassy, fatty and spicy series, increasing them over time. However, the greatest effect was observed in the fatty series through greater extraction of octanoic acid.

The supra-extraction technique led to an increase of fruity and floral series compared to the control, with the floral series reaching the highest levels in SUPRA wines. Terpene compounds are the main contributors to this series, highlighting linalool and β-citronellol besides subthreshold compounds, such as linalool oxide, nerol and α-terpineol. Other studies on Palomino fino have highlighted the neutral character of this variety and its low potential to produce varietal aromas [2,5,8,55]. These results show supra-extraction as a technique capable of selectively extracting terpenes and aromatic precursors from the Palomino fino grape, highlighting its varietal character. 

Regarding the yeast strains used, the results were similar, but slight differences were observed in the fruity, floral and fatty series. The CK strain enhanced the floral series, followed by F and ENSIS, while PC was characterized by its higher levels of the dried fruit series. 

The β-glycosidase enzymes showed a different effect on the principal aromatic series. Both enzymes decreased the ΣOAV of the fruity series, with a greater effect of 12G observed. Likewise, the floral series decreased with the use of this enzyme but increased when the AR enzyme was used, except in the PC wines. This enzyme was characterized by producing higher levels of citronellol.

Therefore, the ΣOAV results indicate that the vinification technique used can improve the fruity and floral characteristic in Palomino fino wines and even highlight varietal aromas.

### 3.3. Principal Component Analysis of Odour Activity Values 

The OAVs, related to the aromatic profile of the wine, were used as variables to carry out the principal component analysis (PCA) (Table 4). Three principal components that accounted for 75.1% of the total variance of the data were extracted by PCA. PC1 (44.7% of the total variability) was mainly affected, with positive values, by acids, amyl alcohol, isoamyl alcohol, 1-octanol and 2-methoxy-4-vinilphenol. This factor represents the effects of winemaking techniques on the fruity and fatty series. PC2 was positively affected by terpenes, heptanoic acid, ethyl butyrate and isoamyl acetate, which explained 29.9% of the total variability between the samples. This component shows the main effects that techniques had on the floral series, directly related to varietal character. 

**Figure 3:**
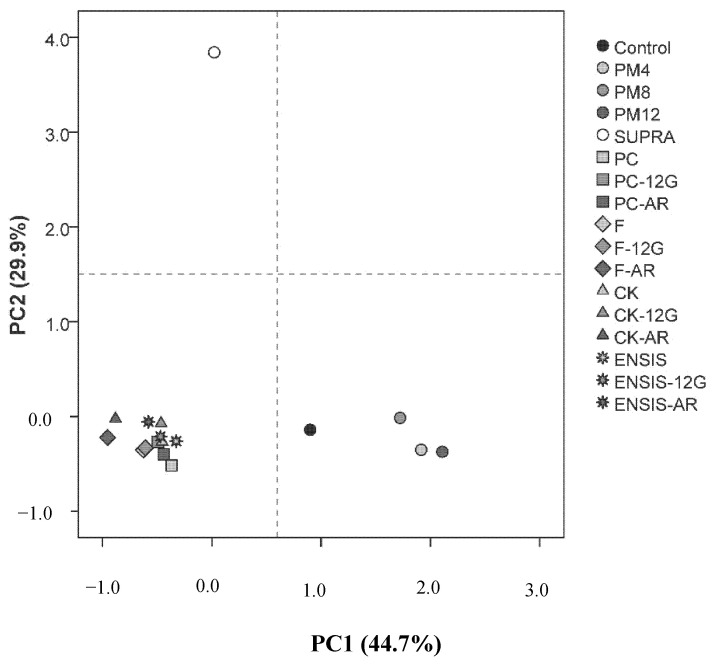
PCA scores of Palomino fino wines elaborated using different vinification techniques. PM4, PM8 and PM12: pellicular maceration for 4, 8 and 12 h, respectively; SUPRA: supra-extraction wine; PC, F, CK and ENSIS: wines elaborated by different yeast strains; 12G and AR: correspond to enzymatic extracts used.

It is clearly seen from the score plot of the samples on the plane defined by PC1 and PC2 (Figure 3) that three types of wines are identified according to their aromatic profile: firstly, there are the closely grouped wines resulting from the use of yeast strains and glycosidase enzymes (negative values of PC1 and PC2); secondly, slightly separated, there are the control and PM wines (positive values of PC1 and negative values of PC2); thirdly, there are the SUPRA wines (negative values of PC1 and positive values of PC2).

The loadings of each compound on the principal components clearly show that terpenic compounds are mainly responsible for the grouping of the floral wines. Fatty acids and their esters are responsible for the differentiation of other wines due to their fruity character, separating those made using strains and enzymes from the control and those from the pellicular maceration. 

### 3.4. Sensory Analysis

**Figure 4:**
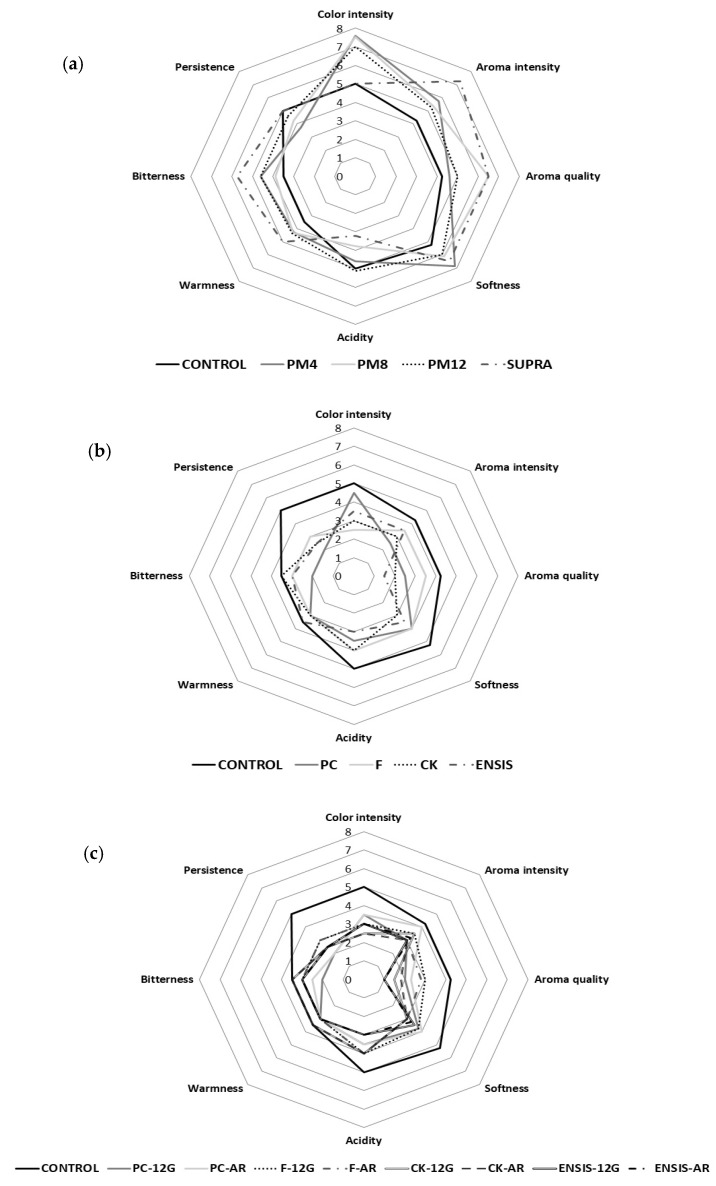
Effect of winemaking technique on sensorial analysis: results of Palomino fino wines compared to the control wine. (**a**) Extractive techniques of pellicular maceration (PM) and supra-extraction (SUPRA); (**b**) aroma-release technique using yeast strains (PC, F, CK and ENSIS); (**c**) aroma release-technique using the β-glycosidase enzymes AR and 12G.

The spider plots (Figure 4) show the mean values for the attributes of the control and treated wines with different winemaking techniques. In general, the wines vinified by extractive techniques (Figure 4a) showed the highest scores for most attributes, especially intensity and aroma quality, indicating an improvement over the control and other wines. As shown in the sensorial analysis, SUPRA Palomino fino wines obtained higher values in the global judgment and were evaluated as superior in terms of aromatic and taste quality. In addition, the tasters valued them as more floral and, above all, fruity, with notes of pineapple, pear, plum and other aromas, such as caramel and raisins, which is consistent with the results obtained in the aromatic profile (OAVs) of these wines. The results described in this study show that the supra-extraction-treated wines had a higher free aromatic profile in comparison to the control wines, mainly due to extraction and hydrolysis of the glycosidically bound terpenes.

On the other hand, the grape origin and the way of obtaining the must have a greater influence on the sensorial profile than the use of yeast strains (Figure 4b) and glycosidase enzymes (Figure 4c), with clear differences in the analysed attributes compared to the control. F wines were the best valued for their aromatic quality with fruity and spicy notes, while PC wines were valued as sherried wines mainly due to their aroma of green apple, nuts and almond, characteristic of the biological aging of sherry wines. This result confirms that obtained in the OAV analysis and explains why the PC yeast strain is selected for the fermentation of Palomino fino musts and its subsequent aging under biofilm of “flor” velum.

The β-glycosidase enzymes led to a significant loss of aromatic quality, resulting in wines classified as “lacking typicality” by the tasters.

## 4. Conclusions

The current study was the first to investigate the influence of different pre-fermentation (pellicular maceration and supra-extraction), fermentation (use of yeasts) and post-fermentation (use of β-glycosidase enzymes) treatments on the volatile compounds, aromatic profile and sensorial evaluation of Palomino fino wines. The magnitude of analytical and sensorial differences observed depended on the origin of the grape variety and the type of press and technique used.

Until now, the Palomino variety has been considered as neutral, with few remarkable aromas, in which the fruity character slightly increases after certain treatments, such as cold skins, fermentation by co-inoculation or the use of glycosidase enzymes. However, the supra-extraction technique showed an increase of volatile compounds in Palomino fino wines and ΣOAVs, improving their aromatic intensity and quality with a greater floral character.

According to the results, supra-extraction is a viable alternative to Palomino fino white wine elaboration, favouring their differentiation from the sherry wines and other white wines made with the same variety.

## Figures and Tables

**Table 1:** Volatile compound concentrations (µg/L) of Palomino fino wines made using extractive techniques.

Volatile Compounds	ID	Control	PM4	PM8	PM12	SUPRA
*Higher alcohols*						
*n*-Propyl alcohol	ST	8134.00 ± 162.68 a	18,214.70 ± 910.70 b	17,998.00 ± 413.95 b	19,415.70 ± 100.96 b	29,103.90 ± 203.72 c
Isobutanol	ST	11,320.00 ± 396.20 a	31,725.00 ± 1078.65 b	29,327.10 ± 1055.77 b	31,000.10 ± 314.21 b	43,641.30 ± 916.47 c
Amyl alcohol	ST	14,982.50 ± 419.49 a	43,114.80 ± 776.05 b	39,081.50 ± 172.43 c	43,057.80 ± 516.69 b	33,019.80 ± 363.22 d
Isoamyl alcohol	ST	65,148.30 ± 1280.18 a	183,491.00 ± 1853.31 b	172,004.20 ± 1408.06 c	176,007.70 ± 893.09 d	149,989.00 ± 1109.92 e
Total		99,585.00 ± 1394.32	276,545.50 ± 1021.46	258,410.80 ± 1065.09	269,481.30 ± 1481.87	244,760.90 ± 1223.77
% Higher alcohols		77.34	87.11	87.24	88.79	85.73
*Methanol*	ST	25,827.00 ± 371.50 a	46,562.50 ± 744.99 b	51,314.00 ± 451.56 c	58,670.90 ± 557.37 d	46,998.00 ± 831.98 b
Total		25,827.00 ± 371.50	46,562.50 ± 744.99	51,314.00 ± 451.56	58,670.90 ± 557.37	47,998.00 ± 831.98
% Methanol		20.1	14.7	17.3	19.3	16.5
*Acids*						
Hexanoic acid	ST	392.03 ± 4.37 a	431.49 ± 10.49 a	381.09 ± 2.42 a	423.36 ± 14.36 a	320.34 ± 10.14 b
Heptanoic acid	ST	3.48 ± 1.14 a	5.55 ± 0.39 b	4.88 ± 0.27 b	4.90 ± 0.17 b	5.36 ± 0.26 b
Octanoic acid	ST	971.99 ± 30.35 a	993.69 ± 25.31 a	967.12 ± 3.75 a	984.04 ± 35.21 a	526.09 ± 63.05 b
*n*-Decanoic acid	ST	27.99 ± 10.67 a	128.59 ± 7.21 b	46.12 ± 4.01 a	153.78 ± 11.76 b	26.93 ± 0.15 a
9-Decenoic acid	LB	52.84 ± 15.49 a	156.62 ± 12.49 b	124.73 ± 10.37 b	287.84 ± 25.14 c	2.00 ± 0.19 d
Benzoic acid	ST	241.76 ± 30.88 a	54.45 ± 7.52 b	42.99 ± 3.73 b	53.62 ± 3.83 b	nd
Total		1690.08	1770.38	1566.92	1907.56	880.73
% Acids		1.32	0.56	0.53	0.63	0.30
*C6 alcohols*						
1-Hexanol	ST	110.10 ± 6.26 a	92.66 ± 0.25 a	100.42 ± 0.52 a	103.75 ± 0.09 a	27.83 ± 2.08 b
(E)-3-Hexen-1-ol	ST	0.96 ± 0.11 a	0.50 ± 0.02 b	0.51 ± 0.21 b	0.52 ± 0.01 b	0.25 ± 0.10 c
(Z)-3-Hexen-1-ol	ST	6.95 ± 0.71 a	5.11 ± 0.26 b	4.49 ± 0.13 b	4.81 ± 0.35 b	4.83 ± 0.30 b
Total		118.02	98.27	105.42 ± 7.09	109.07 ± 15.46	32.91 ± 5.69
% C6 alcohols		0.09	0.03	0.04	0.04	0.01
*Alcohols*		-	-	-	-	-
4-Methyl-1-pentanol	ST	1.78 ± 0.24	1.31 ± 0.19	1.47 ± 0.12	1.35 ± 0.14	1.64 ± 0.22
3-Methyl-1-pentanol	LB	0.71 ± 0.16 a	4.42 ± 0.17 b	4.82 ± 0.06 b	4.71 ± 0.11 b	3.43 ± 0.38 c
1-Octanol	ST	3.18 ± 0.22 a	2.73 ± 0.14 b	2.65 ± 0.12 b	3.03 ± 0.18 a	2.86 ± 0.57 ab
Phenylethyl alcohol	ST	1457.09 ± 35.53 a	1286.68 ± 15.32 b	938.30 ± 43.57 c	1218.67 ± 58.06 d	519.80 ± 83.55 e
Total		1462.77	1295.14	947.24	1227.75	527.73
% Alcohols		1.14	0.41	0.32	0.41	0.18
*Terpenes*						
Linalool	ST	nd	nd	nd	nd	47.20 ± 9.44
Linalool oxide	LB	nd	nd	nd	nd	5.67 ± 0.92
α-Terpineol	ST	nd	nd	nd	nd	26.09 ± 5.22
β-Citronellol	ST	4.12 ± 0.31 a	4.31 ± 0.29 a	2.90 ± 0.58 b	4.38 ± 0.64 a	25.38 ± 3.01 c
Nerol	ST	nd	nd	nd	nd	10.10 ± 2.02
2,6-Dimethyl-3,7-octadien-2,6-diol	LB	24.35 ± 0.73 a	23.97 ± 1.05 a	19.71 ± 0.25 b	22.93 ± 0.10 c	119.76 ± 13.49 d
Total		28.48	28.29	22.61	27.31	234.20
% Terpenes		0.02	0.01	0.01	0.01	0.08
*Esters*						
Ethyl acetate	ST	8023.04 ± 180.71 a	19,107.14 ± 114.64 b	18,988.94 ± 170.90 b	18,179.64 ± 363.59 c	20,899.73 ± 285.98 d
Ethyl butyrate	ST	0.53 ± 0.07 a	0.12 ± 0.05 b	0.51 ± 0.03 a	0.56 ± 0.12 a	2.53 ± 0.28 c
Isoamyl acetate	ST	23.49 ± 2.97 a	11.74 ± 0.02 b	27.21 ± 2.34 a	20.04 ± 1.53 c	73.80 ± 7.11 d
Hexyl acetate	ST	2.61 ± 0.38 a	2.93 ± 0.56 a	4.38 ± 0.36 b	2.89 ± 0.34 a	0.98 ± 0.23 c
Ethyl lactate	ST	1.47 ± 0.55 a	0.82 ± 0.36 a	0.91 ± 0.23 a	1.15 ± 0.35 a	4.25 ± 0.39 b
Ethyl 2-hydroxy-4-methyl butyrate	LB	5.56 ± 0.20 a	5.73 ± 0.23 a	5.32 ± 0.16 a	6.63 ± 0.56 a	3.94 ± 0.23 b
Ethyl 2-hydroxy-4-methylpentanoate	LB	6.83 ± 0.52 a	5.67 ± 0.50 a	5.50 ± 0.47 a	6.03 ± 0.29 a	nd
Ethyl pentanoate	ST	168.91 ± 9.81 a	156.03 ± 4.45 a	118.64 ± 19.92 b	147.11 ± 3.47 a	99.34 ± 9.84 b
Ethyl hexanoate	ST	19.76 ± 2.19 a	19.46 ± 0.58 a	36.33 ± 2.79 b	31.25 ± 2.56 b	41.78 ± 3.11 c
Isoamyl hexanoate	LB	nd	0.16 ± 0.07	0.19 ± 0.10	0.24 ± 0.03	nd
Ethyl octanoate	ST	117.89 ± 7.73 a	157.57 ± 4.45 b	238.61 ± 23.05 c	257.98 ± 26.93 c	140.58 ± 3.21 a
Diethyl malonate	LB	0.16 ± 0.03	0.19 ± 0.01	0.19 ± 0.02	0.18 ± 0.01	0.20 ± 0.05
Ethyl decanoate	ST	21.21 ± 4.90 a	27.11 ± 5.34 a	40.20 ± 6.71 b	36.82 ± 7.43 b	22.14 ± 1.13 a
Diethyl succinate	ST	50.81 ± 3.00 a	51.92 ± 3.04 a	44.35 ± 4.55 a	64.81 ± 4.51 b	61.68 ± 1.54 b
Ethyl 9-decenoate	LB	82.77 ± 4.51 a	100.84 ± 4.55 b	108.30 ± 5.63 b	106.09 ± 4.52 b	31.56 ± 7.24 c
2-Phenethyl acetate	ST	83.66 ± 6.09 a	83.81 ± 7.26 a	80.55 ± 2.00 a	69.25 ± 6.58 b	50.31 ± 4.72 c
Ethyl cinnamate	ST	4.80 ± 0.98 a	11.31 ± 1.79 b	nd	4.72 ± 0.99 a	nd
Total		8613.52 ± 167.40	19,742.53 ± 137.83	19,700.13 ± 111.62	18,935.38 ± 139.41	21,432.81 ± 181.00
% Esters		6.72	6.20	6.67	6.19	7.29
*Aldehydes*						
Acetaldehyde	ST	17,235.10 ± 172.32 a	17,956.90 ± 538.70 b	15,434.50 ± 134.28 c	11,784.00 ± 400.66 d	17,629.50 ± 458.37 ab
Total		17,235.10 ± 172.32	17,956.90 ± 538.70	15,434,50 ± 134.28	11,784.00 ± 400.66	17,629.50 ± 458.37
% Aldehydes		13.29	5.68	5.08	3.96	5.76
*Thiols*						
3-Methylthio-1-propanol	LB	9.13 ± 1.64 a	6.98 ± 0.82 b	4.90 ± 0.39 c	5.57 ± 0.94 b	3.42 ± 0.60 c
Total		9.13	6.98	4.90	5.57	3.42
% Thiols		0.01	0.01	0.01	0.01	0.01
*Phenols*						
2-Methoxy-4-vinilphenol	ST	5.35 ± 0.46 a	9.01 ± 1.86 b	9.54 ± 2.02 b	13.93 ± 2.93 c	nd
2,6-Di-tert-butyl-4-ethylphenol	LB	9.63 ± 1.02 a	8.39 ± 0.21 a	7.64 ± 0.66 a	8.74 ± 0.92 a	5.77 ± 0.55 b
Total		14.98	17.40	17.18	22.66	5.77
% Phenols		0.01	0.01	0.01	0.01	0.01

nd: not detected. PM4, PM8 and PM12: pellicular maceration for 4, 8 and 12 h, respectively; SUPRA: supra-extraction wine; ID: Identification method; ST: compounds detected using pure standards and with Xcalibur v.1.1. Library Browser; LB: compounds detected using with Xcalibur v.1.1. Library Browser. Different letters indicate significant differences concentration volatile compounds analyzed between for Palomino fino wines studied (*p* < 0.05). Data are expressed as mean ± standard deviation (*n* = 3).

**Table 2:** Volatile compound concentrations (µg/L) of Palomino fino wines made using aroma-release techniques.

Volatile Compounds	ID	F	F-12G	F-AR	PC	PC-12G	PC-AR	CK	CK-12G	CK-AR	ENSIS	ENSIS-12G	ENSIS-AR
*Higher alcohols*		-	-	-	-	-	-	-	-	-	-	-	-
*n*-Propyl alcohol	ST	52,340.00 ± 1046.80 a	55,084.00 ± 550.84 b	36,210.00 ± 784.25 c	54,214.00 ± 231.04 b	52,416.00 ± 314.53 a	51,580.00 ± 191.32 a	45,304.00 ± 214.50 c	44,103.00 ± 119.060 d	45,080.00 ± 165.70 d	51,320.00 ± 231.45 a	51,875.00 ± 182.04 a	40,824.00 ± 189.60 e
Isobutanol	ST	58,108.00 ± 552.03 a	60,110.0 ± 947.20 b	45,408.00 ± 791.05 c	61,320.10 ± 320.01 b	54,220.00 ± 208.64 d	61,000.00 ± 401.08 b	53,986.70 ± 289.56 d	52,216.00 ± 487.33 e	54,254.00 ± 614.90 d	56,512.00 ± 713.25 f	63,024.50 ± 682.78 g	47,600.00 ± 503.44 h
Amyl alcohol	ST	24,024.00 ± 288.09 a	25,050.00 ± 151.50 a	18,860.00 ± 334.12 b	24,977.00 ± 146.51 a	23,740.00 ± 203.60 a	24,070.00 ± 114.58 a	31,278.00 ± 407.82 c	29,998.60 ± 216.94 c	31,065.00 ± 387.23 c	27,048.00 ± 145.66 d	30,300.00 ± 278.19 c	22,090.00 ± 106.07 e
Isoamyl alcohol	ST	85,100.0 ± 1021.20 a	87,960.0 ± 985.14 b	64,150.0 ± 879.03 c	89,103.0 ± 915.74 b	81,500.0 ± 1190.06 d	85,260.0 ± 827.54 a	104,005.0 ± 1198.70 e	101,600.0 ± 998.67 f	101,520.0 ± 1010.50 f	112,107.3 ± 778.38 g	118,910.0 ± 963.21 h	89,610.0 ± 532.17 b
Total		219,00.00	228,000.00	164,410.00	229,00.00	210,00.00	221,91.00	234,000.00	227,000.00	231,520.00	246,000.00	264,000.00	200,000.00
% Higher alcohols		54.68	61.24	45.85	46.22	62.21	41.23	65.58	68.28	65.34	68.03	65.11	63.66
*Methanol*	ST	49,321.00 ± 138.52 a	55,010.00 ± 247.25 b	27,763.00 ± 98.74 c	52,024.00 ± 316.07 d	53,117.00 ± 278.90 bd	54,210.00 ± 179.85 b	49,206.50 ± 204.13 a	54,009.00 ± 405.11 b	51,180.50 ± 180.26 d	53,612.00 ± 713.71 b	50,178.00 ± 227.29 a	42,090.00 ± 462.38 e
Total		49,321.00 ± 138.52	55,010.00 ± 247.25	27,763.00 ± 98.74	52,024.00 ± 316.07	53,117.00 ± 278.90	54,210.00 ± 179.85	49,206.50 ± 204.13	54,009.00 ± 405.11	51,180.50 ± 180.26	53,612.00 ± 713.71	50,178.00 ± 227.29	42,090.00 ± 462.38
% Methanol		12.3	14.8	7.7	10.5	15.6	10.1	13.8	16.2	14.4	14.8	12.4	13.4
*Acids*		-	-	-	-	-	-	-	-	-	-	-	-
Hexanoic acid	ST	134.04 ± 15.85 a	144.67 ± 12.45 a	120.19 ± 14.32 a	85.58 ± 31.20 b	119.98 ± 39.87 a	78.93 ± 21.24 b	155.04 ± 26.41 c	140.79 ± 19.87 a	125.17 ± 23.44 a	135.63 ± 15.67 a	126.79 ± 17.6 a	149.28 ± 21.92 c
Octanoic acid	ST	504.44 ± 50.48 a	569.55 ± 46.78 a	370.83 ± 49.60 b	358.74 ± 80.03 b	441.28 ± 85.61 b	284.07 ± 50.11 c	582.00 ± 65.12 a	507.05 ± 58.70 a	300.36 ± 38.69 c	478.23 ± 60.48 a	410.32 ± 62.11 b	421.82 ± 70.90 b
*n*-Decanoic acid	ST	67.09 ± 28.03 a	62.76 ± 25.10 a	nd	nd	43.98 ± 19.20 a	nd	69.46 ± 18.94 a	21.24 ± 2.09 b	nd	58.87 ± 15.78 a	10.72 ± 0.84 c	53.47 ± 16.74 a
Total		705.57	776.99	491.01	444.32	605.24	363.00	806.50	669.07	425.53	672.72	547.83	624.57
% Acids		0.18	0.21	0.14	0.09	0.18	0.07	0.23	0.20	0.12	0.19	0.14	0.20
*C6 alcohols*		-	-	-	-	-	-	-	-	-	-	-	-
1-Hexanol	ST	132.35 ± 11.05 a	93.96 ± 6.59 b	125.60 ± 8.79 a	96.28 ± 3.20 b	80.81 ± 4.57 c	104.17 ± 6.19 d	121.97 ± 4.98 a	88.62 ± 8.91 b	122.27 ± 8.99 a	94.75 ± 2.83 b	71.50 ± 6.48 c	95.70 ± 5.50 b
(E)-3-Hexen-1-ol	ST	0.43 ± 0.04 a	0.38 ± 0.13 a	0.45 ± 0.08 a	0.13 ± 0.01 b	0.42 ± 0.09 a	0.15 ± 0.03 b	0.52 ± 0.12 a	0.45 ± 0.05 a	0.65 ± 0.17 a	0.40 ± 0.01 a	0.40 ± 0.09 a	0.44 ± 0.07 a
(Z)-3-Hexen-1-ol	ST	6.20 ± 0.10 a	5.24 ± 0.36 b	6.53 ± 0.32 a	3.68 ± 0.06 c	4.91 ± 0.13 b	4.26 ± 0.41 b	5.57 ± 0.18 a	4.86 ± 0.23 b	5.70 ± 0.67 a	4.37 ± 0.50 b	4.38 ± 0.95 b	4.88 ± 0.86 b
Total		138.97	99.57	132.58	100.09	86.14	108.57	128.06	93.93	128.62	99.53	76.28	101.03
% C6 alcohols		0.03	0.03	0.04	0.02	0.03	0.02	0.04	0.03	0.04	0.03	0.02	0.03
*Alcohols*		-	-	-	-	-	-	-	-	-	-	-	-
4-Methyl-1-pentanol	ST	4.62 ± 0.35 a	1.11 ± 0.06 b	4.02 ± 0.12 a	0.44 ± 0.09 c	nd	0.49 ± 0.15 c	nd	nd	nd	1.23 ± 0.11 b	nd	1.29 ± 0.24 b
3-Methyl-1-pentanol	LB	nd	5.18 ± 0.18 a	0.00 ± 0.00	nd	nd	nd	2.43 ± 0.12 b	2.31 ± 0.17 b	2.60 ± 0.22 b	3.35 ± 0.27 c	1.62 ± 0.11 d	3.28 ± 0.37 c
3-Ethoxy-1-propanol	LB	7.88 ± 0.25 a	7.64 ± 0.26 a	nd	4.82 ± 0.41 b	6.86 ± 0.36 c	5.15 ± 0.43 b	3.78 ± 0.20 d	3.31 ± 0.46 d	4.30 ± 0.19 b	6.10 ± 0.18 c	4.99 ± 0.25 b	6.59 ± 0.48 c
3-Ethyl-2-pentanol	ST	nd	7.71 ± 0.31 a	nd	1.56 ± 0.11 b	3.65 ± 0.11 c	nd	4.79 ± 0.18 d	nd	nd	nd	Nd	nd
Benzyl alcohol	ST	19.44 ± 0.19 a	9.63 ± 0.9 b	nd	14.25 ± 0.34 c	15.11 ± 0.40 c	nd	7.56 ± 0.23 d	6.54 ± 0.15 d	nd	9.68 ± 0.30 b	10.66 ± 0.74 b	nd
Phenylethyl alcohol	ST	1249.95 ± 81.23 a	1804.51 ± 41.54 b	1925.52 ± 87.41 b	955.23 ± 34.76 c	1117.23 ± 76.76 a	993.57 ± 35.90 c	1573.88 ± 57.98 d	1448.35 ± 43.68 d	1356.26 ± 59.30 d	1192.92 ± 48.60 a	1204.32 ± 47.69 a	1407.93 ± 45.90 d
Total		1281.90	1835.78	1929.54	976.30	1142.85	999.21	1592.44	1460.51	1363.16	1213.28	1221.59	1419.10
% Alcohols		0.32	0.49	0.54	0.20	0.34	0.19	0.45	0.44	0.38	0.33	0.30	0.45
*Terpenes*		-	-	-	-	-	-	-	-	-	-	-	-
β-Citronellol	ST	12.43 ± 0.69 a	9.41 ± 0.32 b	15.29 ± 1.43 c	6.57 ± 0.36 d	6.03 ± 0.27 d	3.94 ± 0.36	13.24 ± 0.80 a	8.34 ± 0.29 b	18.78 ± 1.06 c	10.14 ± 0.64 b	6.35 ± 0.27 d	17.02 ± 0.84 c
2,6-Dimethyl-3,7-Octadien-2,6-diol	LB	13.04 ± 1.24 a	11.52 ± 3.18 a	11.42 ± 2.20 a	9.05 ± 0.51 b	8.37 ± 0.32 b	8.77 ± 0.22 b	10.49 ± 0.50 ab	10.23 ± 0.45 ab	7.62 ± 0.57 b	8.59 ± 0.30 b	14.54 ± 2.04 a	10.43 ± 0.83 ab
Total		25.47	20.93	26.72	15.62	14.40	12.71	23.73	18.57	26.40	18.73	20.89	27.45
% Terpenes		0.01	0.01	0.01	0.00	0.00	0.00	0.01	0.01	0.01	0.01	0.01	0.01
*Esters*		-	-	-	-	-	-	-	-	-	-	-	-
Ethyl acetate	ST	49,958.0 ± 134.91 a	38,081.0 ± 284.23 b	47,140.4 ± 304.78	63,125.0 ± 150.03	33,080.0 ± 97.95	55,730.0 ± 173.49	40,070.0 ± 171.00	24,328.0 ± 121.10	33,780.5 ± 254.71	26,007.0 ± 86.05	38,040.0 ± 58.24 b	27,635.0 ± 91.79
Ethyl butyrate	ST	1.21 ± 0.14 a	nd	nd	nd	nd	nd	1.03 ± 0.11 a	nd	nd	0.63 ± 0.05 b	nd	nd
Ethyl isovaleriate	LB	2.09 ± 0.23 a	nd	2.09 ± 0.17 a	1.14 ± 0.28 b	nd	1.14 ± 0.16 b	1.89 ± 0.36 a	nd	1.89 ± 0.34 a	0.98 ± 0.17 b	nd	0.98 ± 0.16 b
Isoamyl acetate	ST	nd	nd	nd	0.35 ± 0.02 a	nd	nd	nd	nd	nd	0.09 ± 0.01 b	nd	nd
Ethyl lactate	ST	15.77 ± 0.68 a	17.24 ± 1.84 a	23.19 ± 1.04 b	8.67 ± 0.11 c	20.53 ± 0.87 d	13.16 ± 0.94 e	13.87 ± 0.65 e	19.50 ± 0.58 d	21.12 ± 1.73 d	7.81 ± 0.22 c	16.59 ± 0.75 a	12.31 ± 0.32 e
Ethyl pentanoate	ST	98.32 ± 6.20 a	68.47 ± 4.80 b	85.01 ± 3.10 c	39.23 ± 1.05 d	87.99 ± 2.05 c	35.10 ± 1.45 e	53.44 ± 1.07 f	41.46 ± 1.17 d	44.78 ± 2.99 d	59.99 ± 4.43 f	53.77 ± 1.12 f	66.48 ± 1.19 b
Ethyl hexanoate	ST	14.89 ± 0.75 a	nd	8.69 ± 0.95 b	6.67 ± 0.33 c	nd	5.36 ± 0.11 d	14.42 ± 0.55 a	19.50 ± 0.43 e	3.47 ± 0.17 f	9.98 ± 0.31 b	0.99 ± 0.07 g	1.17 ± 0.19 g
Ethyl octanoate	ST	51.64 ± 1.98 a	15.73 ± 0.84 b	43.70 ± 1.75 c	31.30 ± 0.97 d	20.31 ± 0.76 e	24.34 ± 0.76 f	49.94 ± 0.97 a	14.02 ± 0.39 b	55.47 ± 3.08 a	51.26 ± 0.87 a	13.72 ± 0.82 b	46.82 ± 1.44 c
Ethyl decanoate	ST	83.59 ± 2.09 a	32.84 ± 1.15 b	11.43 ± 0.69 c	52.77 ± 1.15 d	30.98 ± 1.09 b	nd	72.10 ± 1.13 e	30.28 ± 0.92 b	27.06 ± 1.86 f	87.42 ± 1.39 a	26.06 ± 0.66 f	25.11 ± 1.38 f
Diethyl succinate	ST	25.66 ± 1.04 a	51.06 ± 1.99 b	41.89 ± 1.12 c	11.77 ± 0.99 d	31.53 ± 0.54 e	19.40 ± 0.32 f	35.57 ± 0.86 g	58.17 ± 1.24 h	60.25 ± 2.55 h	32.63 ± 0.55 e	40.84 ± 0.50 c	53.83 ± 1.67 b
Ethyl 9-decenoate	LB	15.61 ± 0.97 a	10.40 ± 0.32 b	10.72 ± 0.64 b	9.95 ± 0.20 b	9.56 ± 0.58 b	6.11 ± 1.11 c	41.74 ± 0.49 d	17.14 ± 0.78 e	29.62 ± 0.42 f	21.41 ± 0.29 g	7.94 ± 0.69 c	14.57 ± 0.23 a
Phenethyl acetate	ST	57.64 ± 2.04 a	72.58 ± 1.78 b	54.81 ± 3.67 a	38.25 ± 0.67 c	37.73 ± 1.32 c	32.54 ± 1.03 d	97.22 ± 1.00 e	79.47 ± 2.96 f	75.03 ± 0.81 b	76.71 ± 2.50 bf	39.81 ± 1.17 c	71.86 ± 1.36 b
Ethyl palmitate	LB	51.05 ± 0.87 a	nd	nd	nd	90.91 ± 4.98 b	nd	139.40 ± 3.03 c	nd	nd	45.65 ± 0.84 d	nd	nd
Ethyl laurate	ST	nd	nd	nd	nd	nd	nd	23.22 ± 0.77 a	nd	nd	49.40 ± 1.25 b	nd	nd
Ethyl nonadecanoate	ST	25.00 ± 0.27 a	8.97 ± 0.25 b	nd	4.55 ± 0.09 c	nd	nd	nd	nd	nd	nd	nd	nd
Ethyl linoleate	LB	nd	182.53 ± 4.56 a	nd	nd	103.70 ± 2.09 b	nd	49.45 ± 1.03 c	nd	nd	70.57 ± 2.03 d	nd	nd
Ethyl hexadecanoate	ST	nd	36.51 ± 0.75 a	nd	nd	48.25 ± 0.41 b	nd	nd	nd	nd	28.41 ± 0.66 c	nd	nd
Ethyl oleate	LB	nd	90.67 ± 2.05 a	nd	nd	29.29 ± 0.17 b	nd	nd	nd	nd	nd	nd	nd
Diethyl 2-hydroxy-3-methylsuccionate	LB	nd	10.23 ± 0.76 a	nd	nd	10.21 ± 0.14 a	nd	nd	11.77 ± 1.22 a	nd	nd	8.66 ± 0.09 b	nd
Total		50,400.48	38,678.23	47,421.94	63,629.63	33,600.98	55,867.16	40,663.32	24,619.28	34,099.19	26,549.93	38,248.38	27,928.13
% Esters		12.55	10.38	13.21	12.75	9.87	10.38	11.37	7.38	9.61	7.31	9.43	8.88
*Aldehydes*		-	-	-	-	-	-	-	-	-	-	-	-
Acetaldehyde	ST	80,094.0 ± 920.10 a	48,002.00 ± 720.03 b	116,670.0 ± 1,944.5 c	150,315.0 ± 1100.00 d	40,108.50 ± 661.79 e	204,760.00 ± 819.04 f	30,721.0 ± 536.60 g	25,011.0 ± 525.23 h	35,810.50 ± 438.52 i	33,895.5 ± 152.05 j	51,214.0 ± 286.74 k	42,040.0 ± 371.10 l
Total		80,094.0	48,002.00	116,670.00	150,315.0	40,108.5	204,760.00	30,721.0	25,011.0	35,810.5	33,895.5	51,214.0	42,040.0
% Aldehydes		19.95	12.88	32.49	30.20	11.78	38.04	8.59	7.49	10.09	9.34	12.63	13.37
*Thiols*		-	-	-	-	-	-	-	-	-	-	-	-
3-Methylthio-1-Propanol	LB	5.13 ± 0.16 a	7.12 ± 0.29 b	8.75 ± 0.98 c	2.13 ± 0.30 d	5.90 ± 0.99 a	2.06 ± 0.11 d	8.63 ± 0.87 c	8.33 ± 0.56 c	8.87 ± 0.69 c	6.13 ± 0.48 b	6.01 ± 0.76 b	7.73 ± 0.95 b
Total		-	-	-	-	-	-	-	-	-	-	-	-
% Thiols		0.00	0.00	0.00	0.00	0.00	0.00	0.00	0.00	0.00	0.00	0.00	0.00

nd: not detected. PC, F, CK and ENSIS: wine elaborated by different yeast strains; 12G and AR: correspond to enzymatic extracts used; ID: Identification method; ST: compounds detected using pure standards and with Xcalibur v.1.1. Library Browser; LB: compounds detected using with Xcalibur v.1.1. Library Browser. Different letters indicate significant differences concentration volatile compounds analyzed between for Palomino fino wines studied (*p* < 0.05). Data are expressed as mean ± standard deviation (*n* = 3).

**Table 3:** Odour perception threshold (OPT), odour descriptors and odorant series of the volatile compounds found in Palomino fino.

Volatile Compounds	OPT ^a^	Odour Descriptor	Odorant Series
*Higher alcohols*			
n-Propyl alcohol	50	Fresh, fruity	Grassy
Isobutanol	30	Fruity, wine-like	Fruity
Amyl alcohol	30	Fruity	Fruity
Isoamyl alcohol	30	Ripe fruit, sweet	Fruity
*Acids*			
Hexanoic acid	8	Cheese, rancid	Fatty
Heptanoic acid	1	Fatty, dry	Fatty
Octanoic acid	0.55	Vegetable oil, rancid, harsh	Fatty
n-Decanoic acid	1	Fatty, unpleasant	Fatty
9-Decenoic acid	0.04	Waxy, fatty, soapy	Fatty
Benzoic acid	1	Chemical	Chemical
*C6 alcohols*			
1-Hexanol	1.62	Herbaceous, grass, woody	Grassy
(E)-3-Hexen-1-ol	0.4	Fresh	Grassy
(Z)-3-Hexen-1-ol	0.4	Freshly cut grass	Grassy
*Alcohols*			
4-Methyl-1-pentanol	50	Nutty	Dried fruit
3-Methyl-1-pentanol	1	Soil, mushroom	Earthy, mushroom
3-Ethoxy-1-propanol	50	Fruity	Fruity
1-Octanol	0.12	Intense citrus, roses	Fruity
3-Ethyl-2-pentanol	n.f.	-	-
Benzyl alcohol	0.9	Fruity, blackberry	Fruity
Phenylethyl alcohol	10	Roses, honey, lilac, floral, pollen	Floral
*Terpenes*			
Linalool	0.006	Floral, rose	Floral
Linalool oxide	0.006	Fresh, sweet, floral	Floral
α-Terpineol	0.25	Floral	Floral
β-Citronellol	0.018	Floral	Floral
Nerol	0.015	Floral	Floral
2,6-Dimethyl-3,7-octadien-2,6-diol	n.f.	-	-
*Esters*			
Ethyl acetate	12	Pineapple	Fruity
Ethyl butyrate	0.020	Sour fruit, apple	Fruity
Ethyl isovaleriate	0.003	Fresh fruit, orange, berry, blackberry	Fruity
Isoamyl acetate	0.03	Banana	Fruity
Hexyl acetate	0.02	Pear	Fruity
Ethyl lactate	150	Fruity, buttery	Fatty
Ethyl 2-hydroxy-4-methylbutanoate	0.126	Pineapple, strawberry, tea, honey	Fruity
Ethyl 2-hydroxy-4-methylpentanoate	0.051	Blueberry, valerian oil aroma	Fruity
Ethyl pentanoate	1	Apple	Fruity
Ethyl hexanoate	0.014	Green apple, fruity	Fruity
Isoamyl hexanoate	n.f		
Ethyl octanoate	0.005	Pineapple, pear, sweet	Fruity
Diethyl malonate	n.f.		
Ethyl decanoate	0.2	Sweet fruity, dry fruit	Fruity
Diethyl succinate	1.2	Fruity, melon	Fruity
Ethyl 9-decenoate	0.1	Rose	Floral
Phenethyl acetate	0.25	Floral, roses, honey	Floral
Ethyl cinnamate	0.0011	Balsamic, fruity, honey	Fruity
*Aldehydes*			
Acetaldehyde	100	Bitter almond	Dried fruit
*Thiols*			
3-Methylthio-1-propanol	1	Earthy, onion, garlic	Earthy
*Phenols*			
2-Methoxy-4-vinilphenol	0.04	Cloves, spice	Spicy

n.f.: not found. ^a^: Odour Perception Threshold (OPT) (mg/L) and odour descriptors reported by other authors [22,25,26,27,28,29,30].

**Table 4:** Loadings of principal components of volatile compounds’ OAVs in Palomino fino wines.

Volatile Compounds	PC1	PC2
*n*-Propyl alcohol	−0.820	−0.214
Isobutanol	−0.768	−0.539
Amyl alcohol	0.671	0.146
Isoamyl alcohol	0.781	0.272
Hexanoic acid	0.919	0.303
Heptanoic acid	0.365	0.657
Octanoic acid	0.924	−0.035
*n*-Decanoic acid	0.692	−0.125
9-Decenoic acid	0.912	−0.127
Benzoic acid	0.515	−0.074
1-Hexanol	−0.117	−0.710
(E)-3-Hexen-1-ol	−0.695	−0.214
(Z)-3-Hexen-1-ol	-0.247	−0.174
1-Octanol	0.881	0.292
Benzyl alcohol	−0.426	−0.269
Phenylethyl alcohol	−0.272	−0.556
Linalool	0.006	0.990
Linalool oxide	0.006	0.990
α-Terpineol	0.006	0.990
β-Citronellol	-0.507	0.662
Nerol	0.006	0.990
Ethyl acetate	−0.631	−0.283
Ethyl butyrate	0.169	0.797
Ethyl isovaleriate	−0.560	−0.227
Isoamyl acetate	0.090	0.951
Hexyl acetate	0.943	0.058
Ethyl lactate	−0.814	−0.324
Ethyl 2-hydroxy-4-methylbutyrate	0.951	0.227
Ethyl 2-hydroxy-4-methylpentanoate	0.934	−0.124
Ethyl pentanoate	0.809	0.094
Ethyl hexanoate	0.972	0.570
Ethyl octanoate	0.908	0.219
Ethyl decanoate	−0.065	−0.188
Diethyl succinate	0.309	0.388
Ethyl 9-decenoate	0.950	−0.12
Phenylethyl acetate	0.357	−0.131
Ethyl cinnamate	0.739	−0.131
Acetaldehyde	−0.442	−0.256
3-Methylthio-1-propanol	0.064	−0.448
2-Methoxy-4-vinilphenol	0.963	−0.130

Loadings of rotated component matrix. Quartimax with Kaiser normalization of principal component analysis (PCA) of volatile compounds in Palomino fino wines.

## Data Availability

Data is contained within the article.
